# Latest updates on cellular and molecular biomarkers of gliomas

**DOI:** 10.3389/fonc.2022.1030366

**Published:** 2022-11-08

**Authors:** Maroun Bou Zerdan, Ali Atoui, Ali Hijazi, Lynn Basbous, Reine Abou Zeidane, Saada M. Alame, Hazem I. Assi

**Affiliations:** ^1^ Department of Internal Medicine, State University of New York (SUNY) Upstate Medical University, Syracuse, NY, United States; ^2^ Hematology-Oncology Division, Internal Medicine Department, American University of Beirut Medical Center, Beirut, Lebanon; ^3^ Department of Pediatrics, Faculty of Medicine, Lebanese University, Beirut, Lebanon

**Keywords:** gliomas, biomarkers, circulating tumor cells, circulating tumor DNA, immune microenvironment

## Abstract

Gliomas are the most common central nervous system malignancies, compromising almost 80% of all brain tumors and is associated with significant mortality. The classification of gliomas has shifted from basic histological perspective to one that is based on molecular biomarkers. Treatment of this type of tumors consists currently of surgery, chemotherapy and radiation therapy. During the past years, there was a limited development of effective glioma diagnostics and therapeutics due to multiple factors including the presence of blood-brain barrier and the heterogeneity of this type of tumors. Currently, it is necessary to highlight the advantage of molecular diagnosis of gliomas to develop patient targeted therapies based on multiple oncogenic pathway. In this review, we will evaluate the development of cellular and molecular biomarkers for the diagnosis of gliomas and the impact of these diagnostic tools for better tailored and targeted therapies.

## 1 Introduction

Gliomas are central nervous system (CNS) tumors arising from glial or glial precursor cells, mostly localized to the supratentorial region of the brain. Gliomas constitute 30% of all newly diagnosed CNS tumors and up to 80% of malignant CNS tumors and are the biggest contributors to mortality (1). Current standards in the management of gliomas include surgical resection followed by radiotherapy (RT) and alkylating chemotherapy with temozolomide (TMZ). Unfortunately, this aggressive regimen is rarely curative, particularly for higher grade gliomas such as glioblastoma (GBM), the most diagnosed malignant brain tumor. The 5-year relative survival for patients diagnosed with GBM during the 2009-2015 interval ranged from 3% in adults aged ≥ 65 years to 27% among those aged 20-39 years (2).

Classically, glioma classification was based on histological findings and auxiliary tests such as immunohistochemistry (IHC). With the emergence of clinically relevant molecular biomarkers over the past two decades, there has been a shift in the paradigm of classification of gliomas towards an integrated histopathological and molecular diagnosis (3). This change in approach highlights how gliomas that are virtually identical under the microscope may have different molecular signatures that confer different clinical outcomes. Molecular biomarkers were first introduced into the classification of gliomas in the 2016 World Health Organization Classification of Tumors of the CNS (WHO 2016). The value of molecular biomarkers is even more evident in the 2021 WHO CNS5, the current international standard for glioma diagnosis. The WHO CNS5 classifies gliomas into six major families: adult-type diffuse gliomas, pediatric-type diffuse low-grade gliomas, pediatric-type diffuse high-grade gliomas, circumscribed astrocytic gliomas, glioneuronal tumors, and neuronal tumors (4). An array of molecular biomarkers are included in the new classification, including: IDH mutation status, codeletion of chromosomal arms 1p and 19q (1p/19q codeletion), O6-methylguanine-DNA methyltransferase (MGMT) promoter methylation status, epidermal growth factor receptor (EGFR) amplification, telomerase reverse transcriptase (TERT) promoter mutations, H3F3A alterations, nuclear alpha-thalassemia/mental retardation X-linked syndrome (ATRX) gene mutations, loss of cyclin-dependent kinase inhibitor 2A (CDKN2A), combined gain of chromosome 7 and loss of chromosome 10 (7+/10-) and others (4). These markers have been shown to have significant prognostic and predictive clinical value on patient survival, which was the basis of their incorporation into the classification. Additional changes in the WHO CNS5 include the use of Arabic numerals instead of Roman numerals for grading and the incorporation of grading within rather than across tumor types.

Given the limited efficacy of current standards of therapy in gliomas, multiple studies and clinical trials over the past decade have shifted to targeted therapy as an alternative with p53, retinoblastoma (RB), EGFR, FGFR and the proteasome being a few examples (5). Most of these studies showed relatively limited improvements in patient outcomes, partly due to the complexity of the regulatory networks involved. Other studies focused on immunotherapy targets and the tumor microenvironment given their success in certain tumor types.

The recent developments in the field of glioma diagnosis and therapy, coupled with the explosion of cancer genomics and the implementation of new techniques such as liquid biopsy and epigenetic profiling, have led to an evident increase in research focusing on identifying key molecular biomarkers in gliomas. In this review, we will highlight the most recent emerging cellular and molecular biomarkers in gliomas that may provide diagnostic, prognostic, and therapeutic implications and guide future research in this field.

## 2 Discussion with biomarkers

### 2.1 Nuclear and cytoplasmic biomarkers

A growing number of studies are investigating new nuclear and cytoplasmic biomarkers involved in gliomas. For instance, Fatty Acid Binding Protein 7 (FABP7) is highly expressed and localized to the nuclei of IDH1-wt compared to IDH-mut gliomas. Moreover, FABP7-wt overexpression increased cell proliferation rates as well as caveolin-1 expression and caveolae formation through an identified epigenetic mechanism (6). Another emerging biomarker is ribosomal-protein S27 (RPS27), part of the human ribosome 40S subunit that localizes to the cytoplasm and nucleus. RPS27 is overexpressed in many tumors, but its role in CNS tumors such as gliomas wasn’t elucidated until recently. Analysis of healthy, inflamed, neurodegenerative, and cancer brain tissues using IHC, and mRNA sequencing revealed that RPS27 was expressed in all neurons examined and in astrocytic tumor cells but not in normal astrocytes. Interestingly, CD68/RPS27 double staining indicated that almost all macrophages in tumor tissue were positive for RPS27 compared to a minority in inflammatory tissue. Although RPS27 expression levels did not affect patient survival, their association with tumor cells and tumor-associated macrophages (TAMs) provides a rationale for future diagnostic and therapeutic interventions (7). Another relevant nuclear biomarker is rho-specific guanine-nucleotide exchange factor, PLEKHG5, as its expression levels were associated with higher glioma grades. For instance, GBM samples had a higher ratio and stronger intensity of PLEKHG5 expression compared with LGGs. Increased expression level of PLEKHG5 correlated with poorer prognosis and shorter survival time in all glioma patients, suggesting that this nuclear biomarker can have significant prognostic value (8). Likewise, the ETS transcription factor ELK3 was also recently identified as a novel oncogene in gliomas. ELK3 was overexpressed in gliomas compared with normal brain tissue based on database analysis. Moreover, increased ELK3 expression in clinical samples of glioma was associated with reduced overall survival at the 1-, 3- and 5-year intervals. Further studies revealed that ELK3 knockdown decreased the proliferation and migration of a glioma cell line *in vitro*, highlighting the role of this marker in the pathogenesis of glioma (9).

Cytoskeletal elements may also serve as prognostic biomarkers in gliomas. One example is Myosin binding protein H (MYBPH), which was first identified as a myofibrillar component of skeletal and cardiac muscles. MYBPH was overexpressed in GBM tissues based on database analysis, which was further confirmed by IHC in clinical specimens from GBM patients. Moreover, the expression of MYBPH was correlated to IDH mutation and 1p/19q codeletion status. In the IDH-wt and 1p/19q non-codel groups, the expression of MYBPH increased from LGG to HGG in the datasets. The lowest level of MYBPH expression was observed in the IDH-mut and 1p/19q codeletion groups (LGG), while the highest level of expression was observed in the IDH-wt group (GBM) (10). A recent study employed mass spectrometry based proteomic analysis on tumors with known IDH and 1p/19q codeletion status to identify potential surrogates that may be detectable through IHC. Two cytoskeletal proteins, HIP1R and vimentin, were identified as relevant markers that could distinguish between oligodendroglioma and astrocytoma. High HIP1R and low vimentin levels were observed in oligodendroglioma compared to low HIP1R and high vimentin levels in astrocytoma. IHC for HIP1R and vimentin could predict 1p/19 codeletion status accurately in more than 90% of all cases. Adding IHC for ATRX, the only established surrogate marker for a non-1p/19q-codeleted status, increased the sensitivity to 95% (11). Given that identifying 1p/19q status is needed to distinguish between IDH mutant astrocytoma and oligodendroglioma, and the high cost of genetic testing needed to identify it, the HIP1R/vimentin/ATRX approach could serve as an easy and reliable surrogate in clinical practice.

Recent work identified a possible novel tumor suppressor gene involved in gliomas: B-cell leukemia protein 7 family (BCL7). The level of BCL7A expression was significantly lower in glioma tissues compared to healthy brain tissue, and its expression was negatively correlated with glioma grade. Moreover, BCL7A was an independent prognostic factor of LGG and GBM and could predict longer survival in GBM patients receiving TMZ and radiotherapy (12). Another positive prognostic gene in gliomas recently identified is phosphoserine aminotransferase 1 (PSAT1). Particularly, overexpression of PSAT1 predicts a favorable outcome in LGG patients. Interestingly, the combination of overexpression of PSAT1, IDH1 mutation and chromosome 1p/19q codeletion could have the best overall survival in LGG (13).

Even the ubiquitin-proteasome system, which has been a target of cancer therapy in the past years, has been linked to gliomas. A newly identified gene, proteasome 26S subunit ATPase 2 (PSMC2), has been linked to the pathogenesis of multiple cancers, including gliomas. Through broad-spectrum screening of several tumors, PSMC2 was upregulated in most of them, but it was most significantly overexpressed in gliomas and correlated with poor prognosis in glioma patients. Additionally, knockdown of PSMC2 in a glioma cell line inhibited proliferation and affected apoptosis, supporting it as a relevant tumor biomarker (14).

### 2.2 Transmembrane Proteins

Transmembrane protein 158 (TMEM158) has been shown to be significantly upregulated in primary glioblastoma (GBM) compared to WHO grade II or III gliomas based on multiple cancer database analyses. Furthermore, the expression of TMEM158 was higher in IDH1-wt glioma samples compared to IDH1-mut irrespective of grade, and increased expression was correlated with poor OS in glioma patients. Further investigations revealed that TMEM158 enhanced glioma cell proliferation, migration, and invasion as well as the progression of epithelial mesenchymal transition (EMT) by activating STAT3 signaling *in vitro* as well as in a mouse model (15). TMEM158 has been recently implicated in the carcinogenesis of multiple cancers, including gliomas, and more studies are needed to elucidate its exact mechanisms for possible future therapeutic targeting. A similarly conducted study also found that TMEM60 promotes glioma cell proliferation, migration, and invasion and impairs cell apoptosis *via* activating the PI3K/Akt signaling pathway (16). Moreover, translocation associated membrane proteins (TRAMs), which are involved in the posttranslational processing of secretory proteins and translocation to the endoplasmic reticulum (ER) membrane have been implicated in the oncogenesis of gliomas. A recent study found that TRAM2 is over-expressed in glioma samples and cell lines, and that higher expression was associated with poor survival. The researchers further demonstrated that silencing of TRAM2 blocked the malignant progression of glioma by inhibiting the PI3K/Akt/mTOR signaling, rendering it a pathway with therapeutic potential (17).

**Figure 1:**
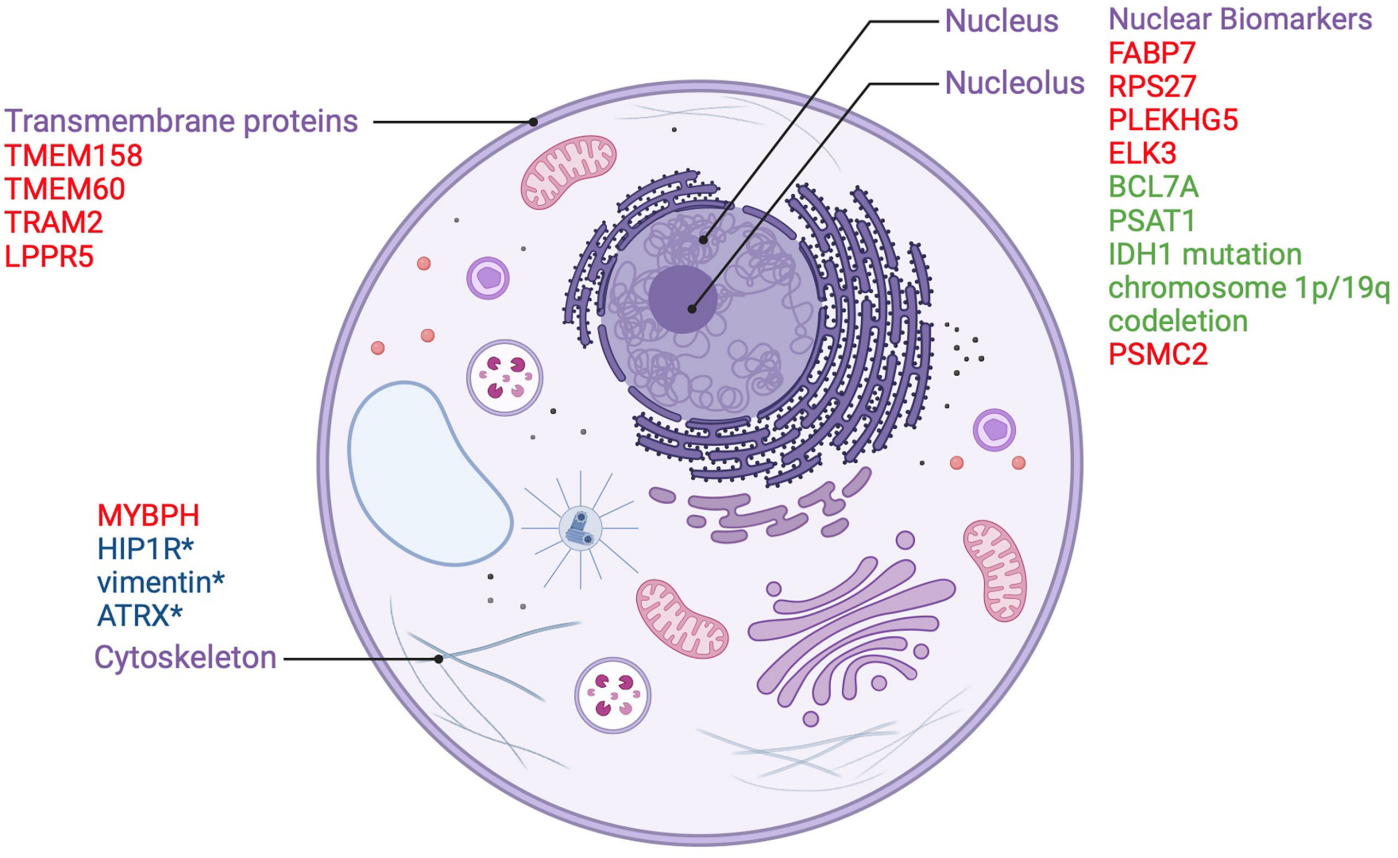
Nuclear and transmembrane biomarkers as prognostic factors in gliomas. Biomarkers in red are poor prognostic factors. Biomarkers in green are good prognostic factors.*used to distinguish between oligodendroglioma and astrocytoma.

Some transmembrane proteins may also play a protective role in gliomas by virtue of their regulatory mechanisms on oncogenic signaling cascades. Lipid phosphate phosphatase-related protein type 5 (LPPR5) which modulates the Rho-GTPase pathway involved in cancer growth, vascularization, and the response to changes in the microenvironment, has been identified as a candidate protective integral membrane protein. Researchers developed a murine orthotopic allograft glioma model with an LPPR5 overexpression model (LPPR5OE) and discovered that LPPR5OE tumors exhibited a more benign phenotype evidenced by delayed growth, increased tumor cell apoptosis, reduced vascular endothelial growth factor A (VEGFA) secretion, and a dysfunctional vascular architecture. Interestingly, the altered architecture showed enhanced susceptibility to sunitinib therapy in the study (18). Despite the novelty of this murine model, the results of this study highlight LPPR5 as a key protein in glioma that warrants further investigation. Nuclear and cytoplasmic biomarkers along with transmembrane proteins which act as prognostic indicators in gliomas are summarized in **Figure 1**.

### 2.3 Immune and immune-microenvironment biomarkers

Gliomas, in theory, should be suitable candidates for targeted immunotherapy, given that immune cells can freely cross the blood brain barrier. However, several immunotherapy trials over the past two decades have shown limited results (5). One of the possible explanations for this phenomenon is the scarcity of tumor infiltrating lymphocytes in gliomas and the abundance of immunosuppressive myeloid cells, rendering them “immune-cold” tumors (19). Aiming to better elucidate the immune microenvironment to identify better immunotherapy targets, a group of researchers performed an integrated analysis of 201,986 human glioma, immune, and other stromal cells at the single cell level. Five specific myeloid cell subtype gene signatures (MC2–MC5, and MC7) were independent prognostic indicators of glioma patient survival, independent of established covariates of glioma patient survival such as IDH mutation and MGMT methylation status. This is a new theme of prognostic markers in gliomas and highlights the value of studying the immune microenvironment. In the study, a candidate gene, S100A4, expressed on immunosuppressive macrophages and T-cells, was significantly associated with poor prognosis in glioma and GBM patients. Moreover, knockout S100a4−/− glioma-bearing mice lived significantly longer than wild-type host mice, validating the potential of S100A4 as an immunotherapy target in GBM (20).

PD-L1 has been long recognized as an immunotherapy target, as it is known to suppress T-cell activity and facilitate cancer progression. In fact, targeting the PD-1/PD-L1 pathway to activate the immune response is an FDA-approved treatment approach for several types of cancer. However, the applicability of this immunotherapeutic modality in gliomas has been limited (5). A recent study utilized transcriptomic analysis to model gene regulation networks in individual gliomas to identify patterns in PD-1 signaling regulation. The regulation of PD1 signaling was repressed in patients with primary GBM who had a long-term survival, while patients with worse outcomes and those with recurrence had a loss of this repression (21). This provides a novel stratification modality to predict patient prognosis and consolidates prior knowledge on the role of PD-1 in cancers and gliomas. It has been suggested that the interaction between PD-1 and PD-L2 could limit the development of a T-cell response and explain the failure of PD-1/PD-L1 immunotherapy trials in older non-glioma related trials (22). This might as well be applicable to glioma immunotherapy. PD-L2 is a cell surface protein well-known to modulate cancer-associated immune responses and has been recently identified as an unfavorable prognostic marker in gliomas. Using data from CGGA and TCGA, higher expression of PD-L2 was observed in higher glioma grades and IDH-wt gliomas and could predict an unfavorable prognosis of patients independent of other factors such as age, grade, IDH status and 1p/19q status. On the other hand, patients with lower PD-L2 expression levels had better survival (23). Furthermore, PD-L2 is associated with the immune response by regulating T-cell function and cytokine secretion. These recent findings further our knowledge of PD-L2 and may provide important clues for future immunotherapy trials targeting this axis.

Another novel glioma biomarker relevant to the immune microenvironment is replication factor 2 (RFC2), a subunit of the RFC complex that modulates DNA replication and repair. In a German study evaluating RFC2 as a prognostic biomarker in glioma, the RFC2 high expression group had higher proportions of naïve B cells, CD8+ T cells, resting memory CD4+ T cells, M0 macrophages, and M1 macrophages and lower fractions of M2 macrophages, resting dendritic cells, and activated mast cells than the RFC2 low expression group. Additionally, RFC2 had co-expression relationships with recognized immune checkpoint genes, including PD-1, PD-L1, PD-L2, B7-H2, and CTLA4 (24). These findings support RFC2 as a possible immunotherapy target. Likewise, the gene plasminogen activator urokinase receptor (PLAUR) which has been linked to extracellular matrix (ECM) degradation in a multitude of tumors, has been identified as an immunological biomarker in glioma. A study recently found that the infiltration level of CD8+ T-Cells decreased while that of macrophages increased along with the increase of PLAUR expression in glioma samples. The macrophages were found to be of the alternative M2 phenotype, which is associated with an immunosuppressive phenotype (25).

Tumor-associated macrophages (TAMs) which may constitute up to 50% of the tumor microenvironment in gliomas, have been shown to promote numerous tumor-promoting activities such as angiogenesis, enhanced tumor cell migration and invasiveness. One of the highly specific markers in TAMs, CD163, was associated with high enrichment of phenotypes of known malignant molecules, such as IDH-wt status based on TCGA and CGGA database analyses (26). Furthermore, there was high concordance between CD163 and immune checkpoints, including PD-L1, PD-1, TIM-3, LAG-3, B7-H3, and B7-H4, making it a promising biomarker and target for immunotherapeutic strategies. Similarly, the immunomodulatory CD161 was found to be enriched in HGG and IDH-wt gliomas and was an independent prognostic factor for the OS of glioma patients. Furthermore, CD161 was shown to inhibit the cytotoxicity of T-cells in glioma patients (22). These findings suggest that CD161 can serve as a marker for reduced tumor cell immunity and a silenced tumor immune microenvironment in glioma, which could serve as a suitable target for immunotherapy.

### 2.4 DNA methylation

DNA methylation is an epigenetic modification that relies on DNA methyltransferases (DMNTs), preferentially acting on the C-5, N-4, N-6 and N-7 sites of DNA segments (27, 28). Genome-wide DNA methylation profiling has proven to be a robust tool in the analysis of epigenetic changes in many cancers, including gliomas. A Swedish study recently investigated the value of methylation profiling using 166 tumor specimens of diffuse low-grade gliomas (dLGGs) in achieving WHO 2016 classification, predicting patient survival, and providing possible refinement to the classification. In predicting IDH mutational and 1p/19q codeletion status confirmed using standard clinical and molecular techniques (such as IHC and FISH), the sensitivity and specificity of methylation profiling were both 100%. Furthermore, the authors compared the methylation-based classification to the WHO 2016 integrated molecular diagnosis and found that methylation profiling provided similar characterization of dLGG in terms of diagnosis and similar prognostication in terms of patient survival (29). These findings, along with the feasibility of obtaining numerous biomarker information in one analysis, make DNA methylation a promising diagnostic and prognostic tool that may be incorporated into clinical practice.

A recent trend found in the literature is the generation of methylation-based signatures that could predict prognosis in gliomas. For instance, a group of Chinese researchers utilized TCGA methylation data to identify prognostic genes in LGGs. Subsequently, they developed a three gene signature (EMP3, GSX2 and EMILIN3) that can be used as a prognostic indicator for LGG patients (30). The signature was in line with the stratification of grade II and III patients and IDH*-*wt cohorts, which may improve current histology-based tumor classification systems and provide better stratification for future clinical trials. In a similar fashion, a signature based on two-CpG DNA methylation sites in LGGs was generated from cancer databases that was independent of other clinical factors like age, WHO grade, family history of cancer and IDH mutation status. Further analysis indicated that the signature exhibited higher predictive accuracy compared with known biomarkers, which may help improve the risk stratification of patients in the clinic (31).

DNA methylation can also be used to predict the response to different glioma therapies. In a study done by Zhou et. Al, the expression of DNA methyltransferase1 (DNMT1) was found to be low in GBM cell lines resistant to TMZ due to the decreased expression of the miR-20a gene which positively correlated with the degree of sensitivity to TMZ (32). Furthermore, the European Organization for Research and Treatment of Cancer (EORTC) 22033 phase III trial randomized patients to two treatment groups, focal RT, or dose dense TMZ, to compare these treatment modalities and identify putative prognostic and predictive molecular markers (33). There was no significant difference in progression-free survival (PFS) for patients in the two groups; however, in the TMZ-treatment arm, patients with IDH-mt co-deleted tumors did better than the IDH-mt non-co-deleted subgroup. A group of researchers recently analyzed the DNA methylome of DNA Damage Response (DDR) genes as predictors of treatment response in this trial (34). Promoter methylation profiles of four DDR genes were found to be predictive of longer PFS in one of the treatment arms: MGMT, MLH3, RAD21, and SMC4. These findings support established studies on MGMT promoter methylation as predictors of benefit from treatment with alkylating agents in GBM (35), and open further avenues for new therapeutic targets in LGGs.

### 2.5 Histone modification

The alteration of histone proteins exhibits an important aspect in the gene regulation process in patients with GBM. Common modifications include acetylation, methylation, phosphorylation and ubiquitylation (36). In general, histone acetylation increases gene expression, while methylation either downregulates or upregulates expression depending on the protein core of the histone involved (d). An abnormal histone modification process can have a tremendous effect on the upregulation of genes that promote GBM proliferation and propagation and can also contribute to acquiring resistance against certain therapeutic regimens (37–39).

For instance, enhancer of zeste homolog 2 (EZH2), a histone methyltransferase involved in the upregulation of c-MYC (40), was shown to be highly involved the tumorigenesis of GBM and decreased the survival rates (41), therefore representing an important prognostic factor related to the grade of the glioma (42). In this sense, the inhibition of EZH2 negatively impacts the ability of GBM cells to regenerate *in vitro*, downregulates tumorigenesis *in vivo* and increases the sensitivity to radiation therapy (43).

Furthermore, protein arginine methyltransferases (PRMTs) are important enzymes in the histone modification process that disrupt the interaction between proteins and their related downstream cellular signaling. They were proved to increase the tumorigenesis of GBM if aberrantly expressed (44, 45). For example, PRMT1 and PRMT2 are overexpressed in GBM and their depletion was shown to decrease tumor cell proliferation in mouse xenografts (46, 47).

In addition, the lysine demethylases (KDM), also involved in the histone modification process, play an important role in GBM resistance to therapy whereby they alter the regulation of cell death, senescence, and tumorigenesis (48, 49). For instance, KDM5A is known to be overexpressed in GBM cell lines resistant to TMZ, and the knockout of this gene efficiently downregulates tumor proliferation *in vivo* and *in vitro* in these resistant cells in addition to increasing their sensitivity to TMZ (50, 51).

Lastly, the acetylation of histone proteins, tightly regulated by histone acetyltransferases (HAT) and histone deacetylases (HDAC), is crucial in order to maintain adequate gene expression (52). Generally, the overexpression of these genes leads to the development of GBM. For example, the expression of HDAC9 was shown to be highly upregulated in GBM, thereby causing an increased tumor proliferation by activating the transcription coactivator with PDZ-binding motif (TAZ), an important downstream component in the Hippo pathway (53). In this sense, an inhibition of HDAC9 was shown to decrease the expression of TAZ and produce an anti-GBM effect (54).

### 2.6 Chromatin remodeling

Remodeling of chromatin represents the alteration of chromatin into higher order complexes which can impact drug resistance depending on the resulting structures and the accessibility that permits transcription (55). A study by Xiao et al. (x) showed that an up-regulation of chromatin remodeling factor lymphoid-specific helicase (LSH) contributed to the development and progression of gliomas. In addition, it was shown that an increased expression of the transcription factor E2F1 and glycogen synthase kinase-3B correlated with the level of LSH in astrocytomas and GBM, also leading to an increased progression of the disease (56). Furthermore, evidence showed that treatment induced resistance in GBM were mediated by a set of transcriptional events regulated by chromatin remodeling processes. In other words, targeting this machinery through inhibitors like PARP inhibitors in treatment-resistant GBM cells could potentially increase sensitivity response to therapy (57).

### 2.7 Circulating tumor cells

Circulating tumor cells (CTCs) are tumor derived cells that are shed into the bloodstream during tumor formation, growth, or invasion (58). CTCs have been long recognized for their clinical applications in cancer screening, genotyping, monitoring tumor progress, and delivery of individualized treatments. The case is no different with gliomas, as several studies over the past decade have demonstrated glioma derived CTCs in the peripheral blood of patients (59). In a 2014 study by Muller et al., CTCs were detected in the blood of 20.6% of patients with GBM by immunochemical analyses using glial fibrillary acid protein (GFAP) (60). Moreover, the presence of CTCs in peripheral blood was assessed before and after surgical resection. CTCs were detected in 13.4% of patients in both presurgical and postsurgical samples, 7.5% only in postsurgical samples and 6% only in presurgical samples. Another study by Macarthur et al. identified the presence of blood CTCs in 72% of patients with GBM, with a decrease to 8% post radiation therapy, confirming the ability of these cells to cross the BBB (61). More recent research conducted on 13 GBM patients undergoing treatment with a microtubule inhibitor revealed that CTCs can cross the BBB in clusters (62). CTCs may also be useful in tracking responses to therapy in glioma patients. In a retrospective study on 22 patients who underwent tumor resection followed by RT and then developed new enhancing mass lesions on MRI, the CTC count was significantly higher in the tumor recurrence group compared to the tumor necrosis group. ROC analysis showed that a cell count threshold of 2 had 91.2% specificity and 100% sensitivity with AUC = 0.933 to predict tumor recurrence, which were superior to standards of diagnosis such as DSC-MRP and MET-PET (63).

Current detection methods of CTCs rely on specific surface antigens, namely the transmembrane glycoprotein epithelial cell adhesion molecule (EpCAM) that is highly expressed in carcinomas. However, gliomas do not express EpCAM, and hence less specific microfluidic techniques are being utilized for detection (59). This is in part why the application of CTCs in gliomas has been limited. Nevertheless, there have been some promising findings in the development of specific glioma-derived CTC detection techniques in recent years. A novel strategy for glioma CTC capture and detection was recently developed, targeting the cancer-specific glycosaminoglycan structure oncofetal chondroitin sulfate (ofCS) (64). It utilizes recombinant malaria VAR2CSA protein (rVAR2) which can specifically bind to glioma cell lines in a background of normal white blood cells and could be used for magnetic capture and isolation of these cells from whole blood with variable efficiency, reaching up to 75%. Moreover, a trial on blood samples derived from ten glioma patients established proof-of-concept for the identification of glioma CTCs. Chinese researchers recently developed a highly sensitive technique for CTC capture in liquid biopsies using antibody-modified immunomagnetic microspheres (IMs) (65). The clinical applicability of this method was confirmed using a mouse xenograft model and clinical specimens from glioma patients. Another interesting isolation modality is isolating CTCs from glioma patients using human telomerase reverse transcriptase (hTERT) (66). The authors report that the detection rate of this method is the highest reported to date (83.02%) and allowed detection of different pathological subtypes other than GBM. CTCs could be isolated by flow cytometry, which has the added advantage of single-cell molecular analysis, which can provide valuable prognostic and therapeutic information.

### 2.8 Circulating tumor DNA

Circulating tumor DNA (ctDNA) refers to circulating cell-free DNA (ccfDNA) derived from tumor cells thought to arise from apoptosis or necrosis of tumor cells or other excretory mechanisms (59). The BBB, however, is a limitation for the detection of ctDNA, as it leads to lower serum levels compared to other tumors (67). To overcome the low detection thresholds, many studies have opted to use CSF as a source to study ctDNA, as it has shown better sensitivity despite being more invasive (68). After obtaining the samples, two approaches are commonly implemented in the detection of ctDNA: targeted mutational sequencing and whole genome sequencing.

In a study of 419 patients with primary brain tumors, including gliomas, ctDNA was detectable in up to half of the cases (69). This was confirmed with another study using the same technique with a detection rate of around 51% in primary GBM (70). Higher sensitivities in the detection of ctDNA in glioma patients may be achieved using targeted sequencing on CSF samples. For example, analysis of the mutational status of commonly mutated genes in gliomas, including IDH1, IDH2, TP53, TERT, ATRX, H3F3A, and HIST1H3B gene mutations, provides higher sensitivity in detection and can guide diagnosis (71). Furthermore, ctDNA can provide prognostic value in glioma patients. This was highlighted in a study on 85 glioma patients, whereby ctDNA was detected in the CSF of 42 of them. Higher levels of ctDNA were observed in cases of progressive disease, CSF space spread, and larger tumor burden (72). ctDNA can also be used to monitor disease progression in glioma patients. A recent study found that patients with brain tumors, including GBM and metastatic cancer, have a 30-fold increase in ccfDNA compared with healthy individuals (73). Upon intranasal therapy with Peirillyl Alcohol (POH), the mean cfDNA serum levels of patients who survived more than 6 months was significantly lower compared with those that survived less than 6 months (2.7 folds). Interestingly, one of the patients under study with stable disease after 3 years of continuous POH therapy developed an increase in ccfDNA 3 months after treatment discontinuation, which was verified by imaging as tumor progression. This constellation of findings indicates that ccfDNA may serve as a noninvasive prognostic and molecular marker in brain tumors and as a possible screening tool for the early detection of tumor progression. Another utility of ctDNA in the realm of glioma is in selecting candidates for targeted therapy. A recent publication analyzing ccfDNA by whole genome sequencing from 25 GBM patients and 25 healthy controls found several gene-gene fusions which may be targets of specific therapies (74). For instance, KDR–PDGFRA and NCDN–PDGFRA were identified in 44% of all samples, BCR–ABL1 in 8%, COL1A1–PDGFB in 8%, NIN–PDGFRB in 8%, and FGFR1–BCR 4%. These findings raise significant clinical and therapeutic implications given that tyrosine kinase inhibitors are known to target such gene fusion products.

With improvements in the current methods available for detection of ctDNA, the use of this modality in the field of gliomas looks promising (75).

### 2.9 Noncoding RNAs

Non−coding RNAs (ncRNAs) are a class of functional non-protein coding RNAs including microRNAs (miRNAs/miRs), long non−coding RNAs (lncRNAs) and circular RNAs (circRNAs).

#### 2.9.1 miRNAs

The role of miRNAs in gliomas has been subject to extensive study in recent years. This is highlighted in a 2018 meta-analysis which found that the overall sensitivity of miRNAs in the diagnosis of glioma was 85%, specificity was 90%, and AUC was 93% (76). Additionally, some miRNAs may serve as prognostic and therapeutic indicators in glioma.

A representative of the potential utility of miRNAs in gliomas is miR-21. The expression of specific exosomal miRNA such as miR-21 has been shown to be significantly higher in HGGs than in LGGs and controls (77). The diagnostic efficacy of miR-21 as a clinical biomarker in glioma was further consolidated by a meta-analysis showing a pooled sensitivity of 0.82 and specificity of 0.94 (78). In terms of treatment, miR-21 was shown to correlate with the response to chemotherapy. In fact, the downregulation of miR-21 induced a better proapoptotic effect of TMZ in GBM cells (79).

When their targets are implicated in gliomagenesis, miRNAs can be utilized as therapeutic modalities. For example, the oncogene FLOT2, which is a known target of miR-449, was recently shown to be greatly upregulated in glioma tissues and cell lines, and its expression level was associated with tumor stage and size. In a study, miR-449 could bind directly to the 3’UTR of FLOT2 and regulate FLOT2 expression in glioma cells. Moreover, the expression levels of miR-449 in glioma tissue and cell lines was significantly reduced (80). This constellation of findings may nominate miR-449 as a therapeutic tool to halt glioma cell proliferation. Another example is miR-376a whose expression could suppress the angiogenic ability of glioma cell lines *in vitro*, whereas using a miR-376a inhibitor exerted the opposite functions. Additionally, xenografts with ectopic miR-376a expression showed smaller volumes and weights and a slower growth, further highlighting the utility of this miRNA (81). **Table 1** highlights the recent relevant studies on miRNAs as diagnostic, prognostic, or therapeutic biomarkers in gliomas.

**Table 1:** Families and types of gliomas with relevant genetic parameters (82).

Tumor Family	Tumor type	Altered molecular profiles
Adult-type diffuse gliomas	Astrocytoma, IDH-mutant	IDH1, IDH2, ATRX, TP53, CDKN2A/B
	Oligodendroglioma, IDH-mutant, and 1p/19q-codeleted	IDH1, IDH2, 1p/19q, TERT promoter, CIC, FUBP1, NOTCH1
	Glioblastoma, IDH-wildtype	IDH-wildtype, TERT promoter, chromosomes 7/10, EGFR
Pediatric type diffuse low-grade gliomas	Diffuse astrocytoma, MYB- or MYBL1-altered	MYB, MYBL1
	Angiocentric glioma	MYB
	Polymorphous low-grade neuroepithelial tumor of the young	BRAF, FGFR family
	Diffuse low-grade glioma, MAPK pathway-altered	FGFR1, BRAF
Pediatric type diffuse low-grade gliomas	Diffuse midline glioma, H3 K27-altered	H3 K27, TP53, ACVR1, PDGFRA, EGFR, EZHIP
	Diffuse hemispheric glioma, H3 G34-mutant	H3 G34, TP53, ATRX
	Diffuse pediatric-type high-grade glioma, H3-wildtype, and IDH-wildtype	IDH-wildtype, H3-wildtype, PDGFRA, MYCN, EGFR (methylome)
	Infant-type hemispheric glioma	NTRK family, ALK, ROS, MET
Circumscribed astrocytic gliomas	Pilocytic astrocytoma	KIAA1549-BRAF, BRAF, NF1
	High-grade astrocytoma with piloid features	BRAF, NF1, ATRX, CDKN2A/B (methylome)
	Pleomorphic xanthoastrocytoma	BRAF, CDKN2A/B
	Subependymal giant cell astrocytoma	TSC1, TSC2
	Chordoid glioma	PRKCA
	Astroblastoma, MN1-altered	MN1

#### 2.9.2 Long non-coding RNAs

The number of studies evaluating the role of lnRNAs as oncogenic and prognostic biomarkers is rapidly growing (83, 84). However, given the complex nature of lnRNAs, it would be best to approach them from a combined analysis approach. A 2018 meta-analysis showed a significant association between elevated lncRNA expression levels and OS in glioma patients (85). Moreover, lncRNA expression was significantly associated with tumor diameter, grade, and Karnofsky Performance Status Scale. Another 2018 systematic review and meta-analysis investigating 40 studies examining the role of lnRNAs in the clinicopathological features, diagnosis and prognosis of gliomas revealed that urothelial carcinoma associated 1 (UCA1) expression was positively associated with tumor size and WHO tumor grade, and that metastasis-associated lung adenocarcinoma transcript 1 (MALAT1) expression could predict poor OS in patients with glioma (86).

Other studies focused on combinations of lnRNAs that may serve as prognostic signatures. Using survival analysis and the Cox regression model, a group of researchers identified a set of six lncRNAs (AC005013.5, UBE2R2-AS1, ENTPD1-AS1, RP11-89C21.2, AC073115.6, and XLOC_004803) that could stratify GBM patients into high- and low-risk groups with significantly different survival (median 0.9 vs. 1.6 years) (87). In a similar fashion, the UVA8 model was built using the TCGA database based on 8 lnRNAs. UVA8 model successfully stratified patients into high and low risk groups and could predict glioma patient survival independent of age, grade and IDH mutation status (88). Another study identified 10 autophagy-associated lncRNAs (PCBP1-AS1, TP53TG1, DHRS4-AS1, ZNF674-AS1, GABPB1-AS1, DDX11-AS1, SBF2-AS1, MIR4453HG, MAPKAPK5-AS1 and COX10- AS1) which were subsequently used to construct a prognostic signature dividing patients into low-risk and high-risk groups with significantly different survival (89). Even so, lncRNA gene methylation patterns could be used to construct signatures predictive of survival in gliomas (90).

Emerging studies have found several potential therapeutic target lnRNAs. For instance, inhibition of the lnRNA antidifferentiation noncoding RNA (ANCR) could repress invasion, migration, and proliferation, as well as promote apoptosis of glioma cells (91). Another lnRNA, ARST, whose expression is significantly decreased in GBM patients, could serve as a potential therapeutic target. A study found that overexpression of ARST in glioma cells significantly suppressed cell growth, proliferation, migration, and invasion and reduced their tumorigenic capacity *in vivo* through interfering with actin cytoskeleton integrity (92).

lnRNAs have been shown to interact with miRNAs to promote glioma progression. LncRNA NEAT1 can promote glioma cancer progression *via* regulation of both the miR-98-5p/BZW1 and miR-128-3p/ITGA5 axes (93, 94). A growing number of studies has revealed intricate interactions *via* complex lnRNA-miRNA networks. LncRNA PART1 can suppress glioma proliferation and migration *via* miR-374b/SALL1 axis (95), LINC00689 can inhibit glioma tumorigenesis *via* the miR-526b-3p/IGF2BP1 axis (96), GAS5 can alter the EMT process, proliferation, migration, and invasion of glioma cells through miR-106b targeting PTEN (97). Other lnRNA-miRNA interactions have been implicated in resistance to glioma therapy resistance. For instance, over-expression of lncRNA TMEM161B-AS1 promotes resistance to TMZ by sponging hsa-miR-27a-3p (98). Another study found that the interaction between the lncRNA-RMRP/ZNRF3 axis and Wnt/β-catenin signaling regulates TMZ resistance in glioma (99). A recent study even suggested lnRNA MIR155HG may serve as an immunotherapeutic target in glioma (100). **Table 2** focuses on lnRNAs as diagnostic and/or prognostic biomarkers in gliomas.

**Table 2:** Table summarizing the details of recent relevant studies on lnRNAs as diagnostic and/or prognostic biomarkers in gliomas.

Year and Reference	lnRNA	Study Type	Patients and Samples	Correlation
2022 [101]	LINC00565 LINC00641	Diagnostic Prognostic	GBM patients (35) Healthy individuals (15)	Sens: 97%, Spec: 100%, and AUC: 0.994 Sens: 100%, Spec: 93.3%, and AUC: 0.995 Negative OS
2022 [102]	MALAT1 SNGH16	Prognostic	Medulloblastoma (41) Epilepsy specimens (5)	Negative Negative
2022 [103]	LINC01087	Prognostic	Glioma tissue (80) Adjacent tissue (80)	Negative
2022 [104]	DLGAP1-AS1	Prognostic	TCGA and CGGA	Negative
2022 [105]	LINC00265 CIRBP-AS1 GDNF-AS1 ZBTB20-AS4	Prognostic	TCGA (504) CGGA (513)	Negative
2021 [106]	ANRIL SOX9	Diagnostic Prognostic	Glioma (142) Healthy volunteers (120)	Sens: 81.62% and Spec: 90.83% Negative OS
2021 [107]	SBF2-AS1	Prognostic	LGG (524 from TCGA, 431 from CGGA)	Negative
2021 [108]	BLACAT1	Prognostic	Glioma tissue (137) Adjacent tissue (137)	Negative
2021 [109]	LINC00355	Prognostic	Glioma tissue (121) Adjacent tissue (121)	Negative
2021 [110]	CYTOR MIR155HG LINC00641 AC120036.4 PWAR6	Prognostic	CGGA (89 LGG and 92 GBM) TCGA (405 LGG and 136 GBM) Patient samples (38 LGG and 53 GBM)	1-year AUC: 0.72 3-year AUC: 0.92 5-year AUC: 0.90
2020 [111]	FTX	Prognostic	Glioma tissue (187) Adjacent tissue (187)	Negative
2020 [112]	ZNF667-AS1	Diagnostic Prognostic	Glioma tissue (155) Adjacent tissue (155)	Sens: 68.22%, Spec: 84.57%, AUC: 0.8541 Negative OS
2020 [113]	ELF3-AS1	Diagnostic Prognostic	Glioma tissue (182) Adjacent tissue (182)	AUC: 0.8073 Negative OS
2019 [114]	LINC00319	Prognostic	Glioma tissue (72) Adjacent tissue (72)	Negative
2019 [115]	PEG10	Prognostic	Glioma patients (147)	Negative
2019 [116]	LINC01503	Prognostic	Glioma patients (133)	Negative
2019 [117]	PXN-AS1-L	Prognostic	Glioma tissue (177) Adjacent tissue (177)	Negative
2018 [118]	PlncRNA-1	Prognostic	Glioma tissue (104) Adjacent tissue (104)	Negative
2018 [119]	AFAP1-AS1	Prognostic	Glioma tissue (52) Non-tumor controls (5)	Negative
2018 [120]	LINC00961	Prognostic	Glioma tissue (151) Adjacent tissue (151)	Positive
2018 [121]	MRCCAT1	Prognostic	Glioma tissue (103) Normal brain tissue (21)	Negative
2018 [122]	MEG3	Prognostic	Glioma tissue (79) Adjacent tissue (79)	Positive
2017 [123]	UCA1	Prognostic	Glioma tissue (64) Normal brain tissue (10)	Negative
2017 [124]	SNHG1	Prognostic	Glioma (78) Normal brain tissue (12)	Negative
2017 [125]	PVT1	Prognostic	Glioma (80) Normal brain tissue (10)	Negative
2016 [126]	AGAP2-AS1 TPT1-AS1 LINC01198 MIR155HG	Prognostic	Anaplastic glioma from GSE16011 (80)	Negative Positive Negative Negative
2016 [127]	AB073614	Prognostic	Glioma (65) Normal brain tissue (13)	Negative
2016 [128]	ZEB1-AS1	Prognosis	Glioma (82) Normal brain tissue from cerebral trauma/epilepsy (13)	Negative
2015 [129]	MALAT1	Prognosis	Glioma tissue (118) Adjacent tissue (15)	Negative
2015 [130]	BC002811 XLOC_010967 NR_002809	Prognosis	Astrocytoma (90)	Positive Positive Negative

TCGA, The Cancer Genome Atlas; CGGA, Chinese Glioma Gene Atlas; OS, overall survival; Sens, sensitivity; Spec, specificity; LGG, low-grade glioma.

#### 2.9.3 Circular RNA

A growing number of studies have implicated circRNAs in a number of biological processes in gliomas, including cell proliferation, metastasis, angiogenesis and oncogenesis (131). For instance, circXRCC5 was upregulated in glioma tissues and cell lines, and correlated with the poor prognosis of glioma patients (132). Furthermore, knockdown of circXRCC5 blocked cell proliferation, migration, and invasion, but facilitated apoptosis. Other circRNAs may also prevent the proliferation of glioma cells and have a protective value. An example is circDCL1, whereby upregulation of this circRNA through METTL3-mediated m6A modification repressed the malignant proliferation of glioma cells (133). Recent studies also suggest that cicRNAs can affect multiple pathways of gliomagenesis, including the tumor microenvironment. circNEIL3 is one such circRNA that was shown to promote glioma carcinogenic progression *in vitro* and *in vivo* (134). Mechanistically, circNEIL3 stabilizes the oncogenic IGF2BP3 protein by preventing its ubiquitination. Furthermore, circNEIL3 is transferred through exosomes to TAMs, enabling them to acquire immunosuppressive properties.

### 2.10 Recent updates on glioma biomarkers from the WHO CNS5 classification

The recent WHO 5CNS classification is mostly guided by Molecular Biomarkers. **Table 1** summarizes some of the altered molecular profiles in gliomas. For instance, CDKN2A/B is one of the criteria to diagnose high-grade astrocytoma with piloid features alongside a piloid cytology, frequent MAPK pathway gene alterations, loss of ATRX nuclear expression, and a distinct DNA methylation pattern (135). Furthermore, to classify a tumor as oligodendroglioma both the IDH-mutation and 1p/19q codeletion should be identified (82, 135).

For some of the tumors, molecular patterns are required for the diagnosis. The presence of one or more of the following three genetic parameters EGFR gene amplification, TERT promoter mutation and 7+/10–, is required to upgrade astrocytoma, IDH-wildtype to glioblastoma, IDH-wildtype (135, 136). Also, the WHO CNS5 classifies tumors with alterations in H3F3A to the pediatric-type diffuse high grade gliomas family as they have significantly worse outcomes, and tumors with homozygous CDKN2A/B deletion as having the highest malignancy grade in the group of diffuse, IDH-mutant astrocytomas (135, 137).

Moreover, new types of gliomas were introduced depending on different mutations found. For example, diffuse astrocytoma, MYB- or MYBL1-altered belong to the family of pediatric-type diffuse low-grade gliomas and are classified as CNS WHO grade 1. Another example is the diffuse low grade glioma, MAPK pathway-altered having mutations in FGFRs and/or BRAF and morphologically resembling a diffuse glioma (135).

## 3 Prognostic and predictive values of different biomarkers

Many studies showed that H3F3A alterations, TERT promoter mutations, CDKN2A deletion, 7q+/10-, EGFR amplifications are significantly associated with worse overall survival and progression free survival (135, 138–141). Whereas IDH mutations especially when associated with 1p/19q deletions, MYB and MYBL1 mutations, MAPK pathway activation, MGMT promoter methylation and upregulated MN1 have positive overall prognostic values (135, 142–144).

In terms of predictive values, the loss of chromosome 1p/19q, in low grade gliomas responding to Temozolomide, predicts both a persistent chemosensitivity and a favorable prognosis (145). Moreover, in preclinical studies, IDH mutant glioma cell lines showed better response to Poly ADP Ribose Polymerase (PARP) inhibition than cell lines with IDH wildtype (146). In addition, promising efficacy was showed with a new covalent binding EGFR-TKI (tyrosine kinase inhibitors), CM93, targeting EGFR alterations in preclinical trials (147), while other TKIs, in phase II trials, failed to show improvement in patients with non-progressive or recurrent glioblastoma (135, 148). These promising therapies should be studied in clinical settings for a possible better disease control.

## 4 Current Challenges and Future Directions

The field of glioma research has exploded in recent years. Part of this increased interest stems from the urgency to improve the outcomes of glioma patients, as their outlook is still gloomy despite multiple advances in the current treatment regimens. Furthermore, the incorporation of molecular diagnostics in gliomas has provided new paradigms in classification and treatment, as evidenced by the changes in WHO classifications over the years. With the availability of extensive cancer databases and the incorporation of next generation sequencing and genome-wide methylation profiling, the field of glioma research is rapidly expanding and offering promising findings that will contribute to a better understanding of these tumors. As extensively highlighted in this review, a growing number of cellular and molecular biomarkers have emerged with significant clinical relevance. The tumor microenvironment also offers great potential in providing novel immunotherapy targets. And with the enhancements in the techniques of liquid biopsy, numerous avenues of intervention are emerging. Future research should focus on moving these biomarkers from *in vitro* and animal studies to clinical studies to better evaluate their efficacy in gliomas.

## Author contributions

MBZ and AA drafted the manuscript. LB, AA, RAZ, SA, MB and AA contributed to the discussion section. HA conceived the idea for the paper. All authors have read and approved the final manuscript. All authors contributed to the article and approved the submitted version.

## Conflict of interest

The authors declare that the research was conducted in the absence of any commercial or financial relationships that could be construed as a potential conflict of interest.

## Publisher’s note

All claims expressed in this article are solely those of the authors and do not necessarily represent those of their affiliated organizations, or those of the publisher, the editors and the reviewers. Any product that may be evaluated in this article, or claim that may be made by its manufacturer, is not guaranteed or endorsed by the publisher.

## Abbreviations

BBB, blood brain barrier; ccfDNA, circulating cell-free DNA; CNS 2016, World Health Organization Classification of Tumors of the CNS 2016; CTCs, circulating tumor cells; ctDNA, circulating tumor DNA; dLGG, diffuse low-grade gliomas; ECM, extracellular matrix; EMT, epithelial-mesenchymal transition; EpCAM, epithelial cell adhesion molecule; FISH, fluorescence *in situ* hybridization; GBM, glioblastoma; IDH1-mut, IDH-1-mutant; IDH1-wt, IDH-1-wild-type; IHC, immunohistochemistry; LGG, low grade glioma; OS, overall survival; PD-L1, Programmed cell death 1 ligand 1; PD-L2, Programmed cell death 1 ligand 2; PFS, progression-free survival; RT, radiotherapy; TAMs, tumor-associated macrophages; TCGA, the cancer genome atlas; TMZ, temozolomide.
